# Supplementary material for: A UMLS-based spell checker for natural language processing in vaccine safety
Source: BMC Med Inform Decis Mak. 2007 Feb 12;7:3. doi: 10.1186/1472-6947-7-3 (PMC1805499; doi:10.1186/1472-6947-7-3)
Supplement: Additional file 13 — RAP application directory. Sets up directory for RAP (RDF application for PHP) source code [file 1472-6947-7-3-S13.gz › rap/doc/phpdoc/syntax/_syntax_N3Serializer_php.html]

xml version="1.0" encoding="iso-8859-1"?


Docs for page N3Serializer.php


## /syntax/N3Serializer.php

Description

Description |
Classes
| Constants

PHP Notation3 Serializer

This class serialises models to N3 Syntax.

Supported N3 features:

- Using [ ] for blank nodes, or \_: if nescessary
- Literal datytype- and xmlLanguageTag support

Un-supported N3 Features include:

- Reification

**History:**

- 12-06-2004 improved namespace handling added (tobias.gauss@web.de)
- 25-03-2004 Bug in doResource() fixed
- 11-27-2003 fixed problems with detection for using [ ] or \_: for blank nodes.
  Added constant N3SER\_BNODE\_SHORT to disable the automatic detection if bnode is referenced elsewhere
- 11-19-2003 Support for datatypes and language tags added.
- 08-01-2003 Made compatible with new v6 MemModel
- 31-07-2003 Function saveAs added. <radol@gmx.de>
- 07-16-2003 Initial version

TODO: \* added namespace prefixes are persisent...

- filesource: Source Code for this file
- access: public
- version: V0.9.1
- author: Gunnar AA. Grimnes <ggrimnes@csd.abdn.ac.uk>, Daniel Westphal <mail@d-westphal.de>

Classes

Description |
Classes
| Constants

| Class | Description |
| --- | --- |
| N3Serializer | An abstract object. |

Constants

Description |
Classes
| Constants

MAGIC\_STRING = '~~~'
(line 43)

Documentation generated on Thu, 7 Jul 2005 13:42:12 +0200 by phpDocumentor 1.3.0RC3
